# Analysis of antibiotic use and clinical outcomes in adults with known and suspected pleural empyema

**DOI:** 10.1186/s12879-022-07759-8

**Published:** 2022-10-12

**Authors:** Benjamin S. Avner, Anush Ginosyan, James Le, Justin Mak, Zeena Qiryaqoz, Cuyler Huffman

**Affiliations:** 1 Department of Internal Medicine, Western Michigan University Homer Stryker MD School of Medicine, Kalamazoo, United States; 2 Department of Biomedical Sciences, Western Michigan University Homer Stryker MD School of Medicine, Kalamazoo, United States

**Keywords:** Empyema, Pleural effusion, Bacterial infection, Antibiotics, Antibiotic use, Intravenous antibiotics, Anaerobic bacteria

## Abstract

**Background:**

There is not a prevailing consensus on appropriate antibiotic choice, route, and duration in the treatment of bacterial pleural empyema after appropriate source control. Professional society guidelines note the lack of comparative trials with which to guide recommendations. We assessed clinical outcomes in the treatment of known and suspected empyema based upon three aspects of antibiotic use: (1) total duration, (2) duration of intravenous (IV) antibiotics, and (3) duration of anti-anaerobic antibiotics.

**Methods:**

We performed a hypothesis-generating retrospective chart review analysis of 355 adult inpatients who had pleural drainage, via either chest tube or surgical intervention, for known or suspected empyema. The primary outcome variable was clinician assessment of resolution or lack thereof. The secondary outcomes were death within 90 days, hospital readmission within 30 days for empyema, and all-cause hospital readmission within 30 days. Mann-Whitney U test was used to compare outcomes with regard to these variables.

**Results:**

None of the independent variables was significantly associated with a difference in clinical resolution rate despite trends for total antibiotic duration and anti-anaerobic antibiotic duration. None of the independent variables was associated with mortality. Longer total antibiotic duration was associated with lower readmission rate for empyema (median 17 [interquartile range 11–28] antibiotic days in non-readmission group vs. 13 [6-15] days in readmission group), with a non-significant trend for all-cause readmission rate (17 [11–28] days vs. 14 [9–21] days). IV antibiotic duration was not associated with a difference in any of the defined outcomes. Longer duration of anti-anaerobic antibiotics was associated with both lower all-cause readmission (8.5 [0–17] vs. 2 [0–11]) and lower readmission rate for empyema (8 [0–17] vs. 2 [0–3]).

**Conclusion:**

Our data support the premise that routine use of anti-anaerobic antibiotics is indicated in the treatment of pleural empyema. However, our study casts doubt on the benefits of extended IV rather than oral antibiotics in the treatment of empyema. This represents a target for future investigation that could potentially limit complications associated with the excessive use of IV antibiotics.

**Supplementary Information:**

The online version contains supplementary material available at 10.1186/s12879-022-07759-8.

## Background

Limited data exist on the role of antibiotics in the treatment of known and suspected bacterial pleural empyema. Bacterial infection of the pleural space is most often secondary to community-acquired pneumonia, although it may also be caused by aspiration, local spread, superinfection of a post-operative hemothorax or hydrothorax, trauma, esophageal perforation, or malignancy-related obstruction [1, 2]. Unlike most other respiratory infectious disease processes, a drainage procedure is the cornerstone of treatment of infectious empyema, with delay in or lack of adequate drainage being tied to worse patient outcomes and higher mortality [1, 3, 4]. Viable means of achieving source control include percutaneous drainage (via chest tube) and surgical drainage (via a thorascopy or thoracotomy procedure).

In addition to drainage, most providers would subsequently provide a course of antibiotics. However, there is not a prevailing consensus on antibiotic choice, route, and duration in the treatment of empyema. The American Association for Thoracic Surgery (AATS), in their 2017 guidelines, identify a range of 2 weeks to 6 weeks in the literature [3]. These guidelines further suggest, without a citation, that oral rather than intravenous (IV) antibiotics are adequate once source control is achieved and the patient is clinically improving. All of the recommendations made by these guidelines with regard to antibiotic use are identified as “level of evidence” (LOE) grade C, representing expert opinion or case studies with limited high-quality data. Similarly, 2010 guidelines from the British Thoracic Society note the lack of evidence to set a firm duration for antibiotics but suggest, also without a citation, at least 3 weeks, guided by clinical monitoring and with antibiotics being changed from the IV to oral route “when objective clinical and biochemical improvement is seen [2].” More information on optimal antibiotic duration is sorely needed to better balance thorough treatment of infection with the risks of line-associated complications, antimicrobial resistance, and *Clostridioides difficile* infection.

A related incompletely answered question concerns the need for antibiotics with a broad spectrum of activity against anaerobic pathogens (hereafter “anti-anaerobic antibiotics”). To the best of our knowledge, no attempts to directly assess the clinical benefit of anti-anaerobic antibiotics are available. For this reason, it is difficult to definitively comment on the clinical benefit of treating anaerobic flora in cases of empyema in which no anaerobes are identified on bacterial culture. AATS guidelines [3] as well as numerous other authors reviewing the literature [3–6] do specifically recommend the use of anti-anaerobic antibiotics as part of the empiric treatment of empyema, e.g. adding metronidazole to ceftriaxone, even if only an aerobic pathogen is identified. However, empiric anti-anaerobic antibiotics are not universally recommended in cases in which *Streptococcus pneumoniae* is grown on pleural fluid culture [2].

In order to determine whether antibiotic choice and duration might impact clinical resolution, we performed a hypothesis-generating retrospective analysis of outcomes in adult patients who had pleural drainage for known or suspected empyema, focusing on the roles of total antibiotic duration, IV antibiotic duration, and anti-anaerobic antibiotic duration.

## Methods

### Study population

Our study population was adult (age ≥ 18) patients admitted between 2015 and 2020 to hospitals of the Bronson Healthcare Group in southwestern Michigan (including Bronson Methodist Hospital in Kalamazoo and Bronson Battle Creek), as well as Borgess Medical Center in Kalamazoo. An initial list of candidate patients was generated via a data audit based around the following search strings:

- ICD-10 diagnostic code “empyema” [J86.9].
- ICD diagnostic code “pneumonia”/”bacterial pneumonia” [J18.9 or J15 series (J15.0-J15.9)] AND pleural drainage procedure performed during that admission [0B9P, 0B9N, 0BBP, 0BBN, 0BTP, 0BTN, 0W99, and 0W9B series].

Additional patient records were also obtained through patients seen on consult by Infectious Diseases in 2020 and with the assistance of a Pharmacy review that provided a database of patients with positive cultures from pleural fluid specimens.

Subsequently, a single investigator (BA) individually reviewed the charts of all patients to assess appropriateness for inclusion. To qualify, patients needed to undergo either chest tube placement or surgical treatment for drainage of a pleural effusion. The effusion drained was required to meet at least one of the following criteria: positive culture for bacteria, exudative by Light criteria, pH less than 7.2, frankly purulent, or surgical appearance of pleural space consistent with empyema. Exclusion criteria included positive culture for a mycobacterial or fungal organism, documentation by medical providers that a culture-negative pleural effusion was highly unlikely to represent infection, and death or hospice enrollment prior to hospital discharge.

### Independent variables and confounders

Basic information collected included age at the time of admission, sex, presence or absence of diabetes mellitus, presence or absence of a defined chronic lung disease (specifically asthma, chronic obstructive pulmonary disease [COPD], or bronchiectasis), glomerular filtration rate (GFR) at the time of drainage, number of days in which patients who received chest tubes had the tube in place, number of days of antibiotics given prior to drainage (either inpatient or outpatient), and intensive care unit (ICU) admission during the hospital stay. For prespecified subgroup analysis, patients were included in either the chest tube group or the surgery group depending upon the means of pleural drainage, and in either the positive or the negative pleural culture group depending upon whether an organism was identified on bacterial culture of a pleural specimen.

The principal independent variables assessed were total duration of all antibiotics, total duration of IV antibiotics, and total duration of an anti-anaerobic antibiotic. β-lactam/β-lactamase inhibitor combinations (such as amoxicillin-clavulanate, ampicillin-sulbactam, and piperacillin-tazobactam), carbapenems, clindamycin and metronidazole were defined as “anti-anaerobic antibiotics.” For the purposes of these independent variables, the antibiotic start date was considered to be date of drainage (the procedural intervention – either chest tube or surgery – that led to the treatment of empyema), whereas antibiotics given in the absence of established source control were not counted towards these totals. For situations in which multiple chest tubes or surgical interventions were performed over the same admission, the reviewer determined whether there was worsening or previously unaddressed empyema or loculations prior to each intervention, and the date of drainage was defined as the date of the final intervention required to address a previously uncontrolled known or suspected infection. A course of antibiotics was arbitrarily defined to have been continuous if there were no gaps of > 72 h between administrations of an antibiotic, and only continuous courses of antibiotic administration were counted when assessing any durations. When patients were discharged with chest tubes in place or on antibiotics, documentation in both the discharge summary and follow-up clinical notes were used to identify or, if necessary, to estimate the durations of chest tubes and antibiotics. In this way, all antibiotics given in the inpatient setting after drainage and in the outpatient setting after hospital discharge were included in our analysis. If a patient died or was readmitted for empyema, this was defined as the stop date of the original antibiotic course.

### Outcome variables

The primary outcome of interest was clinical assessment of disease resolution. This was determined through review of clinic notes and imaging studies obtained after the patient’s discharge from the index hospital. Factors taken into account in review of clinic notes included provider comment that a patient was doing well, provider documentation that infection was presumed cured, or discharge from a surgical practice. When imaging was available, residual pleural effusion was expected to be described by radiologist as reduced or absent in patients who had achieved clinical resolution. We concluded that a patient had achieved clinical resolution if they had either an encouraging provider assessment or encouraging follow-up imaging after discharge, *and* lacked additional hospitalization for empyema or documentation of concern for recurrent disease over the next year. Secondary outcomes were death within 90 days of hospital discharge, readmission within 30 days of hospital discharge for any cause, and readmission within 30 days of hospital discharge due to empyema. Distinction between empyema-specific and non-empyema related readmission was determined by the investigators based on review of whether an ongoing pleural infection was suspected or proven. Adverse drug reactions (ADR) to antibiotics were also identified and recorded.

### Statistical analysis

SAS v9.4 was used to perform all statistical analysis. Significance was assessed at the α = 0.05 level. Primary and secondary outcomes with regard to each independent variable were analyzed by Mann-Whitney U test. For the primary outcome of clinician-assessed resolution, the test was performed independent of others and no adjustment to significance level was made. For the secondary outcomes, a Bonferroni multiplicity adjustment was applied individually for each of the three secondary outcome tests within the variable of interest. Therefore, the significance level of determination was α = 0.05/3 = 0.0166 for each test. For the 4 prespecified subgroup analyses (each containing 12 individual tests), a Bonferroni multiplicity adjustment was performed individually for each subgroup analysis, and the significance level of determination was therefore adjusted to α = 0.05/12 = 0.0042.

The core analysis was repeated with use of anti-anaerobic antibiotics as a dichotomous variable, defining anti-anaerobic treatment as at least 3 days of therapy after drainage. A χ^2^ test was performed to determine if the proportion of patients who met the specified outcomes differed depending on presence or absence of anti-anaerobic treatment. Multiplicity adjustment as above was implemented and the significance level of determination for the associated tests were set to α = 0.05/4 = 0.0125.

A sample size calculation was performed prior to the study. The pre-analysis estimated reference proportion of clinician assessed resolution of disease was 80%. In order to detect a 10% difference, with 80% power, the calculated number of patients needed was 199. In order to detect a 15% difference, with 80% power, the calculated number of patients needed was 76.

## Results

### Description of study population

355 total patients were found to meet the study criteria and were reviewed. Of this population, 338 had follow-up that enabled us to make an assessment of clinical resolution, and 329 had follow-up that enabled us to determine whether they survived for 90 days after hospital discharge. Demographic data by clinical resolution status are shown in Table 1. No statistically significant differences were noted between patients who clinically resolved and those who did not, although there were trends towards a higher percentage of male patients and of patients with preexisting lung disease in the non-resolved group.

**Table 1:** Demographics of patients by clinical resolution status

Demographic features	Total (n = 338)	Clinically resolved (n = 310)	Clinically not resolved (n = 28)	***p***-value
Age		58.4 ± 15.3	61.0 ± 15.8	0.870
Male sex	209 (61.8%)	187 (60.3%)	22 (78.6%)	0.057
Diabetes	86 (25.4%)	76 (24.5%)	10 (35.7%)	0.193
Lung disease (asthma, COPD, or bronchiectasis)	120 (35.5%)	106 (34.2%)	14 (50.0%)	0.094
Glomerular filtration rate ≤ 60	63 (18.6%)	58 (18.7%)	5 (17.8%)	0.912
Chest tube duration (days; chest tube group only)		8.6 ± 15.2	13.3 ± 16.4	0.201
Duration of antibiotics prior to drainage (days)		6.4 ± 6.5	5.5 ± 6.8	0.487
ICU admission	132 (39.1%)	122 (39.4%)	10 (35.7%)	0.657
**Subgroups**				
Drainage via chest tube alone (without surgery)	192 (56.8%)	173 (55.8%)	19 (67.9%)	0.218
Positive pleural culture	171 (50.6%)	157 (50.6%)	14 (50.0%)	0.904

Values given reflect either mean ± standard deviation or total number of patients (percentage of outcome group)

Table 2 shows organisms identified in pleural fluid cultures. Streptococci other than *S. pneumoniae* were the most commonly isolated organism class, growing in samples from 79 patients out of the 170 whose pleural fluid was culture positive. *Staphylococcus aureus* was the second most common pathogen. *S. pneumoniae* was identified in a total of 14 specimens, and was the only organism to be found exclusively in monomicrobial pleural effusions. A total of 34 pleural fluid cultures grew anaerobes (9.6% of all patients, 24.3% of culture-positive patients), of which 28 were identified in the setting of a polymicrobial infection. *Prevotella* spp. were the most frequently identified anaerobes, although specimens also grew *Fusobacterium* spp. as well as a variety of other anaerobic organisms. Among patients who received anti-anaerobic antibiotics, a further breakdown of whether the anaerobic coverage was provided by a β-lactam (i.e.either a β-lactam/β-lactamase inhibitor or a carbapenem) or by another anti-anaerobic drug class is depicted in **Table S1**. 102 of the 230 patients who received anti-anaerobic antibiotics received β-lactam therapy throughout the duration of their anti-anaerobic treatment, and another 47 received anti-anaerobic β-lactams during part of their anti-anaerobic course.

**Table 2:** Organisms identified in empyema cultures

Organism	Total isolates (355 patients)	Isolates in monomicrobial cultures	Isolates in polymicrobial cultures
*Staphylococcus aureus*	54	45	9
*Streptococus pneumoniae*	14	14	0
Other Streptococci	79	57	22
Enterobacterales	14	8	6
*Enterococcus* spp.	8	4	4
*Pseudomonas aeruginosa*	5	2	3
Other aerobic organisms	15	5	10
**ANAEROBES, all**	34	6	28
*Prevotella* spp.	12	1	11
*Fusobacterium* spp.	8	1	7
*Bacteroides* spp.	3	0	3
*Peptostreptococcus* spp.	2	0	2
Other anaerobes	26	4	22

Numbers above reflect isolates of any organism in all pleural cultures throughout the project (n = 355)

### Primary outcome

With regard to the primary outcome of resolution, 310 patients’ empyema resolved with drainage and the antibiotics provided (91.7%), whereas therapy failed to resolve the empyema in 28 patients. There were trends towards association between likelihood of resolution and both median total antibiotic duration and median anti-anaerobic antibiotic duration, but these trends did not reach statistical significance (Table 3). There was no association between median IV antibiotic duration and likelihood of resolution.

**Table 3:** Primary outcome

	Resolved (n = 310) Mean ± StdDev	Resolved (n = 310) Median (Interquartile Range)	Not resolved (n = 28) Mean ± StdDev	Not resolved (n = 28) Median (Interquartile Range)	***p***-value
Total antibiotic duration (days)	21.5 ± 20.2	17.0 (11.0–28.0)	16.4 ± 14.0	14 (7.0–19.0)	0.071
IV antibiotic duration (days)	12.4 ± 15.0	7.0 (4.0–16.0)	12.6 ± 12.8	9.0 (3.0–17.5)	0.970
Anti-anaerobic antibiotic duration (days)	11.8 ± 13.7	8.0 (0–17.0)	6.9 ± 11.9	3.0 (0–7.0)	0.053

Shown are descriptive statistics with regard to clinical resolution vs. non-resolution for the three independent variables. *p*–values are based upon Mann-Whitney U test

### Secondary outcomes

As an additional means of measuring clinical outcome in relation to antibiotic duration, we assessed 90-day mortality, all-cause 30-day hospital readmission, and empyema-specific 30-day readmission (Table 4). Duration of total antibiotic was significantly associated with lower empyema-specific readmission within 30 days; there was also a non-significant trend towards less all-cause readmission within 30 days. Duration of IV antibiotics was not associated with a difference in secondary outcomes. Duration of anti-anaerobic antibiotics was associated with lower 30-day all-cause readmission and with empyema-specific readmission over the same period. Death occurred within 90 days of discharge in 3.1% of the total population (3.3% of population with 90-day mortality data available), and none of the independent variables was associated with a difference in the death rate.

**Table 4:** – Secondary outcomes

	Survived after 90 days (n = 318)	Died within 90 days (n = 11)	***p***-value	Not readmitted within 30 days (n = 290)	Readmitted within 30 days (n = 49)	*p*-value	Not readmitted for empyema within 30 days (n = 321)	Readmitted for empyema within 30 days (n = 17)	***p***-value
Total antibiotic duration (days)	16 (11–28)	16 (8–24)	0.270	17 (11–28)	14 (9–21)	0.018	**17 (11–28)**	**13 (6–15)**	**0.010**
IV antibiotic duration (days)	7 (4–16)	10 (4–24)	0.669	7 (4–16)	9 (5–18)	0.603	7 (4–16)	8 ( 3–15)	0.391
Anti-anaerobic antibiotic duration (days)	7 (0–16)	3 (1–10)	0.520	**8.5 (0–17)**	**2 (0–11)**	**0.003**	**8 (0–17)**	**2 (0–3)**	**0.003**

Shown are median (interquartile range) with regard to 90-day mortality, and 30-day all-cause readmission, and 30-day empyema-specific readmission among evaluable patients for the three independent variables. *p*–values are based upon Mann-Whitney U test with Bonferroni multiplicity adjustment; due to the multiple variables analyzed together, the significance level for each individual test was defined as α = 0.0166. Statistically significant findings are in **bold** text

### Subgroup and additional analyses

We separately analyzed patients treated with chest tubes from those treated with surgery. In the chest tube group, resolution occurred in 173 of 192 evaluable patients (90.1%). Total antibiotic duration, IV antibiotic duration, and anti-anaerobic antibiotic duration were not associated with resolution, although there was a non-significant trend for the latter comparison (data expressed as median [interquartile range]; resolution group 9 [0–19] vs. non-resolution group 2 [0–7], *p*-value 0.028; remainder of data not shown). Duration of anti-anaerobic antibiotics was associated with both lower all-cause 30-day readmission and lower empyema-specific 30-day readmission (no all-cause readmission 9 [0–19] days, n = 162 vs. all-cause readmission 1.5 [0–5] days, n = 30, *p*-value = 0.001; no empyema-specific readmission 8.5 [0–19] days, n = 180 vs. empyema-specific readmission 0.5 [0–2.5] days, n = 12, *p-*value 0.002; remainder of data not shown). In the surgery group, 137 of 146 evaluable patients resolved (93.8%). There similarly was a non-significant trend towards resolution with longer total and anti-anaerobic antibiotic administration (data not shown). Here, however, readmission rates did not significantly differ based upon total antibiotic, IV antibiotic, or anti-anaerobic antibiotic durations (anti-anaerobic antibiotic duration with no all-cause readmission 7.5 [0–16], n = 128 vs. all-cause readmission 6 [0–15], n = 19, *p*-value 0.670; no empyema-specific readmission 8 [0–16], n = 141 vs. empyema-specific readmission 3 [2–6], n = 5, *p*-value 0.469; remainder of data not shown) with the caveat that there were few readmissions among the surgery group.

We further divided the population based on whether patients had culture-positive empyema or culture-negative exudative pleural fluid. Among those without positive cultures, 153 of 166 patients (92.2%) had resolution of their illness. Despite trends towards higher resolution rates with longer durations of total, IV, and anti-anaerobic antibiotics, none of these differences was statistically significant. Among those with positive culture of pleural fluid, clinical resolution occurred in 156 of 170 patients (91.8%), with no association with any of the three independent variables (data not shown).

To further explore the importance of including anti-anaerobic antibiotic therapy in the management of empyema, we dichotomized this variable in order to compare patients who received at least 3 days of anti-anaerobic antibiotics with those who did not (Table 5). Receiving anti-anaerobic antibiotics was significantly associated with a reduced likelihood of being readmitted to the hospital within 30 days, either for any cause or for empyema. The difference was most pronounced for all-cause readmission rates, such that 10.5% of patients who received at least 3 days of anti-anaerobic antibiotics were readmitted within 30 days, compared with 21.9% of those who did not. No association was identified between anti-anaerobic antibiotics and clinical resolution or 90-day survival.

**Table 5:** – Effect of presence or absence of broad anti-anaerobic therapy

	Fewer than 3 days	At least 3 days	***p***-value
Resolution: Yes	106 (89.1%)	204 (93.2%)	
Resolution: No	13 (10.9%)	15 (6.8%)	0.194
90-day death: No	114 (96.6%)	204 (96.7%)	
90-day death: Yes	4 (3.4%)	7 (3.3%)	0.972
30-day readmission: No	**93 (78.2%)**	**197 (89.5%)**	
30-day readmission: Yes	**26 (21.8%)**	**23 (10.5%)**	**0.004**
30-day empyema-specific readmission: No	**107 (90.7%)**	**214 (97.3%)**	
30-day empyema-specific readmission: Yes	**11 (9.3%)**	**6 (2.7%)**	**0.008**

Duration of anti-anaerobic antibiotics was dichotomized to compare via χ^2^ test patients who received 3 days or greater with those who received fewer than 3 days. Data are given as total number of patients and percentage within that treatment group who had each outcome. Statistically significant findings are in **bold** text

### Side effects

Side effects attributed to antibiotics were rare among this study population. A total of 15 out of 355 patients had any antibiotic-associated potential side effect identified in our review (4.2%). Possible ADR included *C. difficile* infection (in 4 patients), drug rash (3), acute kidney injury (3), diarrhea and drug fever (1), leukopenia (1), yeast infection (1), line-associated superficial venous thrombosis (1), and line-associated pulmonary emboli (1). Due to the paucity of patients with side effects, a statistical analysis was not performed.

## Discussion

Despite suggestive trends, our data do not demonstrate a significant difference in clinician-assessed resolution of empyema based on the duration or type of antibiotics used. However, hospital readmission rates support the notion of better outcomes among patients who received longer courses of antibiotics directed broadly against anaerobic pathogens. These effects were most pronounced in the subgroup of patients treated with chest tubes. Our data also indicate that patients who received longer courses of antibiotics were less likely to be readmitted for empyema. However, in no population did the duration of IV antibiotics correlate with the measured clinical outcomes.

For the most part, outcomes were quite good for the patients in our population. Treatment failure occurred in 8.3% of evaluable patients, whereas hospital readmission and generally failure of treatment are reported to be above 10% or higher in some surveys [5, 7]. 90-day mortality in our study was quite low compared to that of some other studies [6, 8, 9]. Given the low number of deaths overall, we presume that our study was underpowered to judge differences in mortality rates between the treatment groups. For similar reasons, as there were relatively few therapeutic failures with any antibiotic treatment regimen, it is difficult to draw many firm conclusions based upon the subgroup analyses. Finally, although we did not directly compare treatment of empyema with chest tube vs. surgical drainage, we note that patients treated with chest tubes had lower rates of resolution and a clearer association between positive outcomes and duration of anti-anaerobic antibiotics.

Aanaerobic bacteria are widely suspected to contribute to disease in empyema. Standard bacterial cultures are reported [2, 4, 5] to be positive in fewer than 60% of cases of empyema. The population of culpable organisms is thought to partially overlap with but to be distinct from the population of organisms that causes bacterial pneumonia, given that pneumonia is not always the cause of empyema and that the relatively hypoxic pleural space is a distinct host environment from the lung parenchyma [5, 6, 10, 11]. Anaerobes are widely agreed to be clinically significant players in cases of lung abscess, necrotizing pneumonia, and bronchopleural fistulas, but some historical and more recent studies have also implicated anaerobes as relatively frequent isolates in cultures of empyema specimens [4, 8, 12–14], and more than 25% of the pleural cultures from empyema patients in Bulgaria in a 2004 investigation grew only anaerobes [14]. In fact, reported rates of isolation of anaerobes may be underestimates of their actual presence given that anaerobes do not always grow reliably in culture, especially in mixed infections and in patients who have already received antibiotics by the time a specimen is obtained for culture. We acknowledge that studies using molecular methods have also supported the premise that anaerobes are present in pleural infections [6]. There has thus been ample reason to suspect that anti-anaerobic antibiotics would provide benefit in this clinical setting, although the above does not provide definitive guidance for antibiotic choice in cases in which only aerobic organisms are isolated in culture. However, it is also worth noting that anaerobes differ in their susceptibility to β-lactam antibiotics. In the study noted above, over 60% of the anaerobic organisms isolated were susceptible to penicillin, and they were nearly universally susceptible to β-lactams with more robust anti-microbial coverage such as ampicillin-sulbactam and cefoxitin [14].

The present investigation joins a relatively limited literature on the topic of the use of antibiotics after drainage. A smaller 2016 study [7] by investigators from Mayo Clinic retrospectively reviewed antimicrobial treatment in 91 patients hospitalized for empyema, almost all of whom underwent surgical treatment and who received 4–29 days of IV antibiotics and 14–30 days of total antibiotics. In this population, shorter courses of both IV antibiotic duration and total antibiotic duration correlated with higher likelihood of treatment failure. Discrepant results between the cited study and the present investigation may reflect differences in patient population, treatment range, or analysis of data, and reinforce the inherent limits of any conclusions that can be drawn from retrospective analyses. The only prospective study in adults of which we are aware randomized a narrowly defined patient population in Spain – non-ICU patients with “complicated” parapneumonic effusions requiring chest tube drainage who were clinically and radiographically recovering at 14 days while on oral amoxicillin-clavulanate – to receive either a third week of antibiotic or placebo [15]. Outcomes were extremely similar between the two groups, with clinician-assessed cure rates at 90 days being 100% in the two-week antibiotic group. Despite the underpowered data, these results certainly suggest that two weeks of antibiotics with early transition to oral therapy may be appropriate for treatment of some pleural effusions including mild empyemas in relatively healthy patients. Generalizability to different empyema scenarios is unclear. Although extensive discussion of empyema in pediatric patients, in whom management recommendations and etiologic organisms may differ compared to adults [16], is beyond the scope of this article, several larger studies have been performed in this population. At least two retrospective studies identified similar or better outcomes in pediatric patients with parapneumonic effusions and/or empyema when they were discharged from the hospital on oral, rather than IV antibiotics [17, 18]. Several pediatric centers have implemented protocols for empyema that include a time-limited course of oral antibiotics after discharge [19, 20].

Our agenda was to shed light on three separate questions with regard to the use of antibiotics in the treatment of known and suspected empyema: (1) optimum duration, (2) indication for IV antibiotics, and (3) indication for anti-anaerobic antibiotics. Based on the above data, with regard to 1), we interpret our results to support the benefits of a longer course of antibiotics. We extrapolate that recommendations suggesting a course of antibiotics of at least three to four weeks are appropriate, barring additional data. This conclusion is based on the fact that patients on longer courses of antibiotics had lower readmission rates for empyema, as well as non-significant trends towards several other favorable outcomes. Although a longer course of antibiotics was not associated here with better odds of resolution or survival, it would be hard to exclude the possibility that a larger study would have detected such an association. As the median duration of therapy in our study was less than 3 weeks (13 days in empyema-specific readmission group, 17 days in group without empyema-specific readmission), further investigation would be required in order to make specific recommendations with regard to optimal antibiotic length.

With regard to 2), we did not demonstrate any evidence that a longer course of IV antibiotics would have any benefit in the treatment of empyema. Especially in light of the suggestive data for total antibiotic duration, the lack of association of IV antibiotic duration with clinical outcome is striking. Prospective investigations directly comparing outcomes in response to IV and oral antibiotics are indicated, as the ability to treat empyema using mostly or entirely oral antibiotics could potentially reduce inpatient stay length, patient morbidity and functional limitation, line associated complications, health care costs, and antimicrobial resistance.

Finally, with regard to 3), our results provide strong support for the premise that anaerobes often contribute to infections of the pleural space. Although the question of whether or not anti-anaerobic antibiotics are required in all cases of suspected empyema was not directly assessed, we did consistently find an association between use of anti-anaerobic antibiotics (such as clindamycin, metronidazole, and penicillin/β-lactamase inhibitors) and lower readmission rates, especially in patients managed with chest tubes rather than surgery. This is certainly in keeping with the existing guidelines that recommend empirically treating for anaerobes in all cases of suspected empyema, with the possible exception of infections caused by *S. pneumoniae*.

Our study is limited by the fundamental limits of retrospective data collection, a strategy that cannot make firm conclusions with regard to causality and is prone to unmeasured confounding factors. Of particular note, clinician prescribing behavior entirely controlled whether a patient received a shorter or a longer course of antibiotics. Numerous patient specific and provider specific factors presumably determined what course of treatment was ultimately agreed upon for each individual patient, and these variables cannot be effectively accounted for in a study based around retrospective chart review. For this reason, the results reported here would need to be confirmed through a prospective study in order to become the basis for any clear-cut recommendations or guidelines. We therefore frame the current work as hypothesis generating rather than definitive. Another limitation of our data is that our definition of cases for inclusion was deliberately broad with regard to the fact that any patient who had a chest tube or surgical procedure for an exudative pleural effusion could potentially be included. This allowed for higher recruitment of patients with an appropriate clinical scenario, doubtlessly including some with non-empyema parapneumonic effusions. It is therefore possible that this study underestimates the duration of antibiotics required to treat empyema due to inclusion of non-empyema cases. Overall, the case definition used here reflects the real-world clinical situation, wherein the diagnosis of culture-negative empyema is not always certain. Finally, we note that our primary outcome variable of “resolution” is inherently subjective and prone to recall and observer bias, so the use of such a variable as the primary outcome represents a key limitation of the study. Of note, the primary outcome appeared to be less sensitive than measurements of hospital readmission for detecting differences in outcomes between groups. We attempted to incorporate a range of outcome variables including nonspecific outcomes that could be clearly defined (mortality, hospital admission) and more subjective outcomes that allowed for more nuanced descriptions (empyema-specific readmission, recovery) in order to provide as complete a description of treatment success as possible within the limits of our study methodology.

## Conclusion

In summary, this retrospective study assessed the relationship between antibiotic selection and duration and the clinical outcomes of adults hospitalized with empyema and exudative pleural effusions requiring drainage. Anaerobes were identified in 24.3% of positive cultures of pleural fluid, and a longer duration of anti-anaerobic antibiotics was associated with better outcomes based on some of the variables investigated, including lower rates of readmission. On the other hand, a longer duration of IV antibiotics had no association with clinical outcomes. Some limited data suggest the possibility of better outcomes with longer total antibiotic duration. We conclude that anaerobes should be empirically treated when designing antibiotic regimens for empyema and possible empyema, and that at least three weeks of total antibiotics should be given. We further conclude that oral antibiotics should be further investigated as a potentially favorable alternative to IV antibiotics in empyema treatment, and we will propose and plan a follow-up prospective investigation to more directly make this comparison.

## Electronic supplementary material

Below is the link to the electronic supplementary material.

Supplementary Material 1

## Data Availability

The datasets generated and analyzed during the current study are available from the corresponding author on reasonable request.

## Declarations

This statement confirms that all methods were carried out in accordance with institutional policies governing human subjects research at Western Michigan University Homer Stryker MD School of Medicine. This information can be accessed on the medical school website.

## Abbreviations

AATS: American Association for Thoracic Surgery
ADR: Adverse drug reactions
COPD: Chronic obstructive pulmonary disease
GFR: Glomerular filtration rate
ICU: Intensive care unit
IV: Intravenous
LOE: Level of evidence
