# Supplementary material for: Analysis of antibiotic use and clinical outcomes in adults with known and suspected pleural empyema
Source: BMC Infect Dis. 2022 Oct 12;22:783. doi: 10.1186/s12879-022-07759-8 (PMC9558363; doi:10.1186/s12879-022-07759-8)
Supplement: Supplementary file 1 — Supplementary Material 1 [file 12879_2022_7759_MOESM1_ESM.docx]

**Table S1 – Antibiotic classes used to treat anaerobic pathogens**

|  | TOTAL PATIENTS | Resolved | Non resolved | Unknown outcome |
| --- | --- | --- | --- | --- |
| β-lactam based (BLB) | 102 | 88 | 11 | 3 |
| Non-β-lactam based (NBLB) | 81 | 74 | 3 | 3 |
| Switched from BLB to NBLB | 27 | 23 | 1 | 3 |
| Switched from NBLB to BLB | 20 | 20 | 0 | 0 |

For the 230 patients who received 3 days or more of anti-anaerobic antibiotics, antibiotics were classified as “β-lactam based” if they included a β-lactam/β-lactamase inhibitor combination or a carbapenem, and “non-β-lactam based” if broad anti-anaerobic coverage was provided only by an antibiotic from another drug class. The number of patients that received each class of anti-anaerobic antibiotics is shown above. Multiple patients were switched from BLB to NBLB or vice versa over the course of their anti-anaerobic treatment.
